# Supplementary material for: Ionic liquid-modified MCM-41-polymer mixed matrix membrane for butanol pervaporation
Source: R Soc Open Sci. 2019 Jul 24;6(7):190291. doi: 10.1098/rsos.190291 (PMC6689585; doi:10.1098/rsos.190291)
Supplement: Data; Supporting information from Ionic Liquid-Modified MCM-41-Polymer Mixed Matrix Membrane for Butanol Pervaporation [file rsos190291supp1.docx]

**Supporting information**

**Ionic Liquid-Modified MCM-41-Polymer Mixed Matrix Membrane for Butanol Pervaporation**

Yifang Li ^a^, Dandan Yan ^b^ and Yanhui Wu ^b *^

(a Shanghai Shenglan Petrochemical Engineering Technology Co. LTD, Shanghai 201200, China

b Shanghai Key Laboratory of Chemical Assessment and Sustainability, School of Chemical Science and Engineering, Tongji University, Shanghai 200092, China

** Author for correspondence: wuyanhui@tongji.edu.cn (Yanhui Wu)*

Data of swelling experiment and pervaporation experiment in this paper are summarized and NMR data of the ionic liquids are provided.

1. **Figure 6**

**Table S1** Effect of time on DS of modified membrane

| Time (h) | DS of PEBA(%) | DS of 5% MCM-41-IL1-PEBA(%) | DS of 5% MCM-41-IL2-PEBA(%) |
| --- | --- | --- | --- |
| 0.5 | 0.16 | 1.24 | 1.21 |
| 1 | 1.31 | 1.71 | 1.81 |
| 1.5 | 1.31 | 2.00 | 2.53 |
| 2 | 1.31 | 2.19 | 3.26 |
| 3 | 1.31 | 2.23 | 3.38 |
| 4 | 1.31 | 2.60 | 3.56 |
| 5 | 1.31 | 2.53 | 3.47 |

DS- degree of swelling

1. **Figure 7 a)**

**Table S2** Effect of MCM-41-IL1 content on DS of modified membrane

| MCM-41-IL1 content (%) | 0 | 2 | 5 | 10 |
| --- | --- | --- | --- | --- |
| DS in water | 0.24 | 0.15 | 0.58 | 0.98 |
| DS in 2.5wt% butanol | 2.09 | 2.41 | 2.60 | 3.59 |

1. **Figure 7 b)**

**Table S3**  Effect of MCM-41-IL2 content on DS of modified membrane

| MCM-41-IL2 content (%) | 0 | 2 | 5 | 10 |
| --- | --- | --- | --- | --- |
| DS in water | 0.24 | 0.10 | 0.31 | 0.86 |
| DS in 2.5wt% butanol | 2.09 | 2.18 | 3.47 | 4.09 |

1. **Figure 10a)**

**Table S4**  Effect of MCM-41-IL1 content on pervaporation performance

| MCM-41-IL1 content (%) | 0 | 2 | 5 | 10 |
| --- | --- | --- | --- | --- |
| Separation factor | 19.45 | 21.47 | 22.72 | 20.02 |
| Permeation flux (g/m^2^⋅h) | 310.14 | 376.11 | 410.00 | 367.22 |

1. **Figure 10b)**

**Table S5**  Effect of MCM-41-IL2 content on pervaporation performance

| MCM-41-IL1 content (%) | 0 | 2 | 5 | 10 |
| --- | --- | --- | --- | --- |
| Separation factor | 19.45 | 22.25 | 25.40 | 21.08 |
| Permeation flux (g/m^2^⋅h) | 310.14 | 390.56 | 421.70 | 376.67 |

1. **Figure 10c)**

**Table S6** Pervaporation performance compare of different membranes

| Membrane | Separation factor | Permeation flux (g/m^2^⋅h) |
| --- | --- | --- |
| PEBA | 19.45 | 310.14 |
| 5%MCM-41 | 19.30 | 408.10 |
| 5% MCM-41-IL1-PEBA | 22.70 | 410.00 |
| 5% MCM-41-IL2-PEBA | 25.40 | 421.70 |

1. **Figure 11a)**

**Table S7**  Effect of temperature on Separation factor

| Temp (^o^C) | Separation factor | | |
| --- | --- | --- | --- |
|  | PEBA | 5% MCM-41-IL1-PEBA | 5% MCM-41-IL2-PEBA |
| 25 | 12.78 | 15.35 | 15.24 |
| 30 | 15.86 | 19.55 | 20.64 |
| 35 | 19.45 | 22.70 | 25.40 |
| 40 | 21.87 | 24.84 | 27.28 |
| 45 | 23.41 | 26.41 | 28.81 |

1. **Figure 11b)**

**Table S8**  Effect of temperature on permeation flux

| Temp (^o^C) | Permeation flux (g/m^2^⋅h) | | |
| --- | --- | --- | --- |
|  | PEBA | 5% MCM-41-IL1-PEBA | 5% MCM-41-IL2-PEBA |
| 25 | 144.0 | 168.8 | 204.2 |
| 30 | 216.1 | 243.5 | 252.2 |
| 35 | 310.1 | 410.0 | 421.7 |
| 40 | 425.6 | 591.7 | 594.4 |
| 45 | 511.0 | 630.0 | 685.0 |

1. **Figure 12**

**Table S9**  Pervaporation performance of 5%MCM-41-IL2-PEBA membrane vs. time

| Time (h) | Separation factor | Permeation flux  (g/m^2^⋅h) |
| --- | --- | --- |
| 0 | 25.4 | 421.7 |
| 10 | 25.1 | 414.1 |
| 24 | 25.2 | 407.1 |
| 48 | 25 | 411.7 |
| 72 | 25.2 | 420.0 |
| 100 | 25.1 | 410.0 |

1. **NMR data of ionic lliquids:**

**IL1, Ionic liquid [EVIM][Tf2N]**

^1^H NMR (600 MHz, Chloroform-*d*) δ 8.78 (d, *J* = 1.7 Hz, 1H), 7.58 (t, *J* = 2.0 Hz, 1H), 7.41 (d, *J* = 1.9 Hz, 1H), 7.00 (dd, *J* = 15.6, 8.7 Hz, 1H), 5.71 (dd, *J* = 15.6, 3.1 Hz, 1H), 5.31 (dd, *J* = 8.7, 3.1 Hz, 1H), 4.19 (q, *J* = 7.4 Hz, 2H), 1.46 (t, *J* = 7.4 Hz, 3H).

^13^C NMR (151 MHz, Chloroform-*d*) δ 133.54 , 127.88 , 122.80 , 120.75 , 119.51 , 118.62 , 109.96 , 45.51 , 14.68 .

^19^F NMR (565 MHz, Chloroform-*d*) δ -79.42.

**IL2, Ionic liquid [OMPY][Tf2N]**

^1^H NMR (600 MHz, Chloroform-*d*) δ 8.82 – 8.72 (m, 2H), 8.48 – 8.42 (m, 1H), 8.00 (t, *J* = 7.1 Hz, 2H), 4.54 (t, *J* = 7.6 Hz, 2H), 1.95 (p, *J* = 7.4 Hz, 2H), 1.33 – 1.14 (m, 10H), 0.80 (t, *J* = 7.0 Hz, 3H).

^13^C NMR (151 MHz, Chloroform-*d*) δ 145.52 , 144.30 , 128.60 , 118.71 , 62.47 , 31.48 (d, *J* = 2.8 Hz), 28.74 (d, *J* = 13.1 Hz), 25.82 , 22.43 , 13.87 (d, *J* = 2.5 Hz).

^19^F NMR (565 MHz, Chloroform-*d*) δ -79.13.
